# Effects of Continuous Yoga on Body Composition in Obese Adolescents

**DOI:** 10.1155/2021/6702767

**Published:** 2021-08-25

**Authors:** Marisa Poomiphak Na Nongkhai, Rodsarin Yamprasert, Chuchard Punsawad

**Affiliations:** ^1^Department of Sports Science and Exercise, School of Medicine, Walailak University, Nakhon Si Thammarat 80160, Thailand; ^2^Division of Applied Thai Traditional Medicine, Faculty of Public Health, Naresuan University, Phitsanulok 65000, Thailand; ^3^Department of Medical Sciences, School of Medicine, Walailak University, Nakhon Si Thammarat 80160, Thailand

## Abstract

Overweight/obesity is a pressing international health concern, and conventional treatments demonstrate poor long-term efficacy. Several studies have shown that yoga can control risk factors for cardiovascular disease, obesity, and psychosocial stress. The present study aimed to assess the effect of continuous yoga (asanas, pranayama, and Surya Namaskar yoga) on body composition in overweight participants. Forty adolescents with obesity were enrolled in this study. The study was conceived as a prospective, single-center, single-blinded randomized controlled trial. The participants were divided into 2 groups: the intervention group (*n* = 20), which undertook a continuous yoga practice, and the control group (*n* = 20). Body composition, including body weight (BW), body mass index (BMI), body fat mass (BFM), and muscle mass, was evaluated using tetrapolar bioelectrical impedance (BIA). Our results showed that the mean BMI and BFM of the yoga intervention group were significantly decreased at week 8 and week 12. The muscle mass of the yoga group continued to improve at a rate of 0.515 per week, which was statistically significant. In conclusion, a continuous yoga practice had a tendency to decrease BMI and BFM and increase muscle mass. These findings demonstrate intervention effectiveness similar to that observed in other clinical research and indicate that continuous yoga practice may be used as an alternative therapy for obesity prevention and health promotion in adolescents with obesity.

## 1. Introduction

Obesity is a metabolic disorder with excessive fat accumulation in different parts of the body, and it is a risk factor for cardiovascular and metabolic disorders [1]. One-third of the world's population is now categorized as overweight, and all signs point to a further escalation of this situation in the years to come [1]. This health problem is increasing worldwide, especially in developing countries and newly industrializing countries [2]. Improvements in living standards and changes in lifestyle, physical inactivity, sedentary behavior, and excessive energy intake have resulted in a rapid increase in overweight and obesity rates among children and adolescents [2–4]. An estimated 38.2 million children under the age of 5 years were overweight or obese in 2019. Once considered a high-income country problem, overweight and obesity are now on the rise in low- and middle-income countries, particularly in urban settings. In Africa, the number of overweight children under 5 has increased by nearly 24% since 2000. Almost half of the children under 5 who were overweight or obese in 2019 lived in Asia. The prevalence of overweight and obesity among children and adolescents aged 5–19 has risen dramatically from 4% in 1975 to just over 18% in 2016. This increase occurred in boys and girls; in 2016, 18% of girls and 19% of boys were overweight [5]. The results of a 2014 nationwide health survey of Thai people found that Thai people over 15 years old were more likely to be overweight and obese (body mass index (BMI) of 25 kg/m^2^ or more) than people who participated in the survey in 2009; the rate among women increased from 40.7% to 41.8%, and the rate among men increased from 28.4% to 32.9% [6]. Moreover, the Department of Health, Ministry of Public Health, Thailand, reported that 13.1% of school-aged children were on the verge of becoming overweight or obese in 2016, and 1 in 4 children and 3 in 4 teenagers were obese and may grow to become obese adults. Obesity increases the risk of developing chronic noncommunicable diseases (NCDs) [7]. College and university students may be especially at risk for sedentary behavior because much of their campus day consists of classroom lectures and studying while sitting still. However, young adults are typically in good health, and educational institutions and student welfare associations facilitate student's engagement in various forms of physical activity. Studies suggested that university students are highly sedentary and highly active [1, 8].

Therapeutic intervention programs for obese individuals aim at long-term weight maintenance and normalization of body weight and body fat. These programs modify the eating and exercise behaviors of obese children and establish new, healthier behaviors and lifestyles. Treatment programs must include behavioral components to permanently change the nutrition and physical exercise habits of obese children and adolescents [9]. All of this information shows that adolescent obesity remains a major public health problem that must be addressed. Control of eating habits and physical activity is recommended in medical guidelines as the most important therapeutic option in nonmorbid obesity [10]. However, a substantial proportion of obese persons not adhering to such recommendations supports the study of alternative forms of physical activity to reduce weight [11]. Yoga is a holistic mind-body intervention aimed at physical, mental, emotional, and spiritual wellbeing. Several studies showed that yoga and/or meditation controlled risk factors for cardiovascular diseases, such as hypertension, type II diabetes, insulin resistance, obesity, lipid profile abnormalities, psychosocial stress, and smoking. Some randomized studies suggest that yoga/meditation retards or even regresses early and advanced coronary atherosclerosis [12]. A recent study suggests that transcendental meditation may be extremely useful in the secondary prevention of coronary heart disease [13] and may decrease body weight [11].

Yoga is one such intervention, with studies reporting long-term adherence and benefits in various health conditions, including obesity. The various postures of yoga, especially forward bending, twisting and backward bending, help reduce fat near the abdomen, hips, and other areas [14]. Therefore, yoga is a solution for a healthy lifestyle because the practice of yoga is a complete package with wonderful cardiovascular, dynamic workouts that do not require any machines or much space [15].

Therefore, yoga may be a way to prevent obesity in adolescents. It is necessary to study forms of exercise that are appropriate and beneficial for weight. The present study examined the effects of a continuous yoga program on weight loss and body composition. This study provides new exercise knowledge on classic yoga plus aerobic exercise for decreasing body weight and increasing fat-burning in female adolescents.

## 2. Materials and Methods

### 2.1. Study Design and Participants

The study was conceived as a single-center, single-blinded randomized controlled trial (RCT) before patients were recruited. All procedures performed in studies involving human participants were in accordance with the ethical standards of the Mae Fah Luang University Ethics Committee on Human Research (MFU EC) at Mae Fah Luang University, Chiang Rai, Thailand (approved no. REH-61202). The total duration of the study was from June 2018 to July 2019.

Forty participants were selected at the beginning of the study and invited to participate in this research. The type of sampling was purposive sampling, which was set up for the main purpose of this study. The selection criteria included (1) female students who studied exercise and weight control as first priority and other subjects who were interested in participating in the research project, (2) age between 19 and 22 years, (3) BMI of 23–29.5 kg/m^2^ and not greater than 30 kg/m^2^, (4) BFM between 26 and 36%, and (5) absence of a disease that could have contributed to obesity (e.g., hypothyroidism and polycystic ovarian syndrome). Participants who had other diseases that were disabling or were not controlled with medication or were participating in another research project were also excluded. Written informed consent was obtained from all participants prior to enrollment in this study.

The sample size determination was calculated from the formula *N* (each group) = (*r* + 1) (*Zα*/2 + *Z*1 − *β*)2 *σ*2/rd2 [14–16], where *Zα* is the normal deviate at a level of significance (*Zα* is 1.96 for 5%) and *Z*1 − *β* is the normal deviate at 1 − *β*% power with *β*% of type II error (0.84 at 80% power). *R* = *n*1/*n*2 is the ratio of the sample size required for 2 groups, which generally creates equal sample sizes for 2 groups. *σ* and *d* are the pooled standard deviation and difference of means of 2 groups, respectively. We performed a pilot study, and the minimal detectable difference of means (d) of two groups was 2.8 scores of body fat mass (BFM), with a standard deviation (*σ*) of 3.00. Therefore, the minimum sample size for each group to detect the mean difference between the two means was 18 persons/group. Considering a 10% of drop-out, twenty patients per treatment group were required for the study.

### 2.2. Continuous Yoga Intervention

Participants were given all of the equipment needed for the yoga classes to use free of charge during the intervention. Equipment included a mat, 2-3 m straps, 3 blankets, and 2 wooden blocks. The intervention was implemented three times per week for twelve weeks, and each class lasted 50 min. The yoga curriculum is shown in Table 1. This curriculum was designed to focus primarily on the physicality of yoga practice. Continuous yoga is a cyclic yoga practice with minimal rest periods. The purpose of this study was to adjust the form of yoga to obtain an aerobic workout and increase the processes used for energy metabolism. Asanas were specifically chosen to strengthen and align the trunk and lower extremities [15–17]. Pranayama, also called breathing exercises, involves manipulation of the breath, which is a dynamic bridge between the body and mind [16–18]. Classes included instructions/demonstrations of each asana, followed by participant practice. Participants reported current pain intensity at the beginning of each class. The instructor actively modified asanas where necessary for pain and/or other limitations (e.g., body habitus) and gave instructions on breathing techniques throughout. The exercise sessions consisted of three phases for a total of 50 mins: 10 min of warming up/stretching in standing poses and a sitting pose, 30 min of continuous yoga involving 12 poses that were performed continuously with no rest or break, and 10 min of relaxation (cooldown) in sitting poses and a corpse pose. Exercise was performed at 65–75% of the maximum heart rate (HRmax). The frequency of exercise was Monday, Wednesday, and Friday from 5 : 00 to 5 : 50 p.m. for 12 weeks. The continuous yoga program, including the asanas technique, was validated by five yoga experts and sports science teachers, and the accepted exercise program was tried out on 20 female students. The continuous yoga practices, which included stretching techniques, sun salutations, and meditation and breathing exercises, were introduced in the yoga curriculum in Table 1.

### 2.3. Assessments of Body Composition

Height was assessed using an FBT height scale. Body composition, including body weight (BW), BMI, BFM, and muscle mass, was measured three times before the intervention (week 0) and at week 8 and week 12 from 4 : 00 to 5 : 00 p.m. Body composition measurement methods are continuously being perfected, with the most commonly used method being bioelectrical impedance analysis (BIA) (INBODY 720 BIOSPACE, USA). Before BIA, participants were instructed to remove their shoes and socks, and the bottoms of the feet and palms of the hands were wiped using tissue. Participants were asked to step onto the BIA device, place the soles of their feet on the foot electrodes, and grip the hand electrodes with their hands. Participants were asked to stand with their arms straight and away from their trunks so they were not touching their bodies to achieve a proper testing posture, as described in the manufacturer's guidelines. The target heart rate (HR) was checked after 30 min of yoga asanas. Participants checked their own HR for 1 min.

### 2.4. Statistical Analysis

All statistical analyses were performed using IBM SPSS® Statistics version 26. The Mann–Whitney *U* test was used to compare the mean values between groups. Friedman's test was used to analyze changes in the mean values from baseline to the 8^th^ and 12^th^ week for each group. Univariable analysis was used to examine the crude odds ratio (COR) of the binary outcome variable for each independent variable. All variables in the univariable analysis were subjected to multivariable analysis to adjust for possible confounders. The final analysis was interpreted as adjusted odds ratios (AORs) with 95% CIs. A *P* value less than 0.05 indicated statistical significance.

## 3. Results

### 3.1. Demographic Characteristics of the Study Participants

Forty participants were initially screened between October 2018 and January 2019. They were studying at Mae Fah Luang University, Chiang Rai Province, Thailand, and were randomized into 2 groups (20 participants in each group). The first group was the yoga or intervention group, and the second group served as the control group. The results demonstrated that the mean weight (kg), BMI (kg/m^2^), BFM, and muscle mass were not different between the control group and the yoga group. The mean height (cm) of the control group was significantly greater than the yoga group (Table 2). There was no significant difference between the two groups in weight, BMI, or BFM. At baseline, the participants in the control group had a lower percentage of body fat and less muscle mass than the participants in the yoga group (Table 2).

### 3.2. Body Mass Index

**Figure 1:**
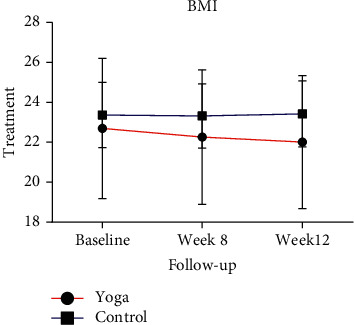
Trends in body mass index (BMI) at baseline, week 8, and week 12 in the yoga and control groups. Data are presented in means (SD).

BMI is a measure of body fat based on height and weight that applies to individuals aged approximately 19–20 years. BMI was calculated based on self-reported body weight (kg) divided by height squared (m^2^) [17–19]. Participants were categorized based on BMI according to World Health Organization Western Pacific Region (WPRO) criteria, as recommended for Asians. BMI was categorized as follows: 23.0–24.9 kg/m^2^ was overweight, 25.0–29.9 kg/m^2^ was obesity level 1, and over 30.0 kg/m^2^ was obesity level 2 [20]. Our study showed that the mean BMI of the yoga group decreased significantly at week 8 and week 12 (Table 3 and Figure 1). However, the mean BMI of the control group did not change compared to the yoga group. When considering differences between the groups, the mean BMI of the yoga group decreased consistently by week 12 (Table 3).

### 3.3. Body Fat Mass

**Figure 2:**
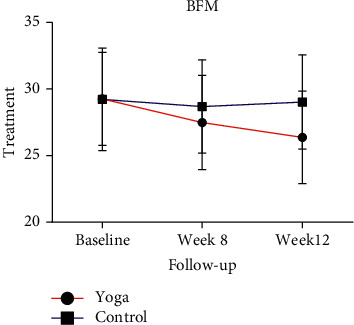
Trends in body fat mass (BFM) at baseline, week 8, and week 12 in the yoga and control groups. Data are presented in means (SD).

The BFM of the yoga group decreased consistently at week 8 and week 12, whereas the BFM of the control group did not change from baseline (Table 4 and Figure 2). At week 12, the mean BFM of the yoga group had consistently decreased compared with that of the control group (Table 4 and Figure 2).

### 3.4. Muscle Mass

**Figure 3:**
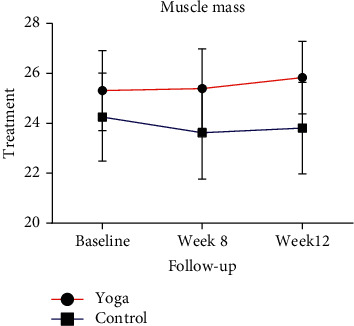
Trends in muscle mass (BFM) at baseline, week 8, and week 12 in the yoga and control groups. Data are presented in means (SD).

The muscle mass of the yoga group was significantly improved at week 8 and week 12, with an improvement rate of 0.515 per week (Table 5 and Figure 3). In contrast, the muscle mass of the control group was significantly decreased compared with the baseline (Table 5 and Figure 3).

### 3.5. Logistic Regression Analysis

The results of the univariable and multivariable analyses of the risk factors associated with overweight are shown in Table 6. Univariable analysis revealed that BFM and muscle mass were significantly associated with overweight (odds ratios (OR) = 1.75; 95% confidence intervals (CI) 1.27–2.71, OR = 4.53; 95% CI 1.75–11.69). No significant associations were found between overweight and age or yoga (*P* < 0.05) (Table 6). Multivariable analysis also showed that muscle mass was significantly associated with overweight. When muscle mass increased by 1 unit, the odds of becoming overweight increased 3.88 times after adjusting for age, BFM, and yoga (adjusted odds ratios (AOR) = 3.88; 95% CI 1.08–13.93) (Table 6).

## 4. Discussion

The prevalence of obesity is increasing worldwide, and obesity is an important risk factor for cardiovascular and metabolic disorders. Particularly worrisome is that children and teenagers with obesity may become obese adults. Obesity is associated with an increased risk of developing chronic NCD. Therefore, alternative therapies for obesity prevention and promotion of health in overweight adolescents are important. The effectiveness of yoga for weight control and improved body composition is evident from surveys [21] and clinical studies [22], and yoga has the potential to increase fat loss, develop muscle tone, and build flexibility, leading to better shape and good proportions. Many types of yoga also help build muscle strength and endurance [23].

The present study assessed the effect of a continuous yoga intervention on body composition in overweight participants. BMI is a parameter of body composition that may be used in the diagnosis of obesity [24]. It is a measure of body fat based on height and weight. Therefore, BMI does not measure body fat directly, but it is moderately correlated with more direct measures of body fat. BMI appears to be strongly correlated with various metabolic and disease outcomes, but there are more direct measures of body fatness [24].

Our results showed that significantly reduced BMI and BFM were found in the yoga group. The Mama study (2019) reported that 12 weeks of yoga led to a significant decrease in body fat. The decrease in body fat may be because the volunteers experienced an abnormal state of yoga exercise over some stretch of time, which produced a decrease in the body fat rate. Yoga includes profound nostril breathing, adaptability of limbs, and extension of various body parts, which may be the reason for the decrease in body fat of the volunteers performing yoga. The decrease in body fat may impact body mass, and the present examination found a significant decrease in body mass in the volunteers performing yoga. Comparable perceptions were noted in numerous studies, where a decrease in body fat was noted after yoga training [23]. A previous study found that an increase in frequency, a longer duration, and the use of complex yoga interventions with multiple components affected anthropometric measures. The combination of yoga with dietary/nutritional recommendations, especially a vegetarian diet with or without calorie reduction, also affected anthropometric measures [25].

The mean BW and muscle mass in the present study were significantly decreased in the yoga group after continuous yoga practice. A controlled trial in India supported that yogic practices contributed to a reduction in excess body fat in school students and obese patients [26]. This finding suggests that yoga plays a role as a safety measure. Participants in the continuous yoga group also had significantly increased muscle mass, which may be due to yoga poses that included strengthening exercises.

Muscle mass includes skeletal muscles, smooth muscles, such as cardiac and digestive muscles, and the water contained in these muscles. Muscles act as an engine in consuming energy. As muscle mass increases, the rate at which energy (calories) is burned increases, which accelerates the basal metabolic rate (BMR) and helps reduce excess body fat and weight in a healthy manner. When exercising hard, muscle mass increases and metabolism is efficient. It is important to monitor body measurements regularly to observe the impact of a training program on muscle mass [27].

A study in Tampere, Finland, also supports the present study and indicated that the practice of yoga was associated with significant decreases in total cholesterol and triglycerides in subjects with cardiovascular disease [28]. A study in Connecticut, USA, used a six-week program of yoga and meditation to observe brachial artery reactivity, and significant reductions in blood pressure, heart rate, and BMI were observed in the cohort that practiced yoga [29]. BW decreased significantly after Hatha yogic practices in a previous study. A controlled trial in India showed that yogic practices contributed to a reduction in excess body fat in school students and obese patients [30]. Ha et al. [31] reported that regular and continuous modified Hatha yoga exercise effectively improved body composition, decreased plasma malondialdehyde (MDA) concentration, and increased plasma superoxide dismutase (SOD) activity in female patients with shoulder pain. Therefore, Hatha yoga exercise should be effective at preventing shoulder pain of various causes in female patients with skeletal muscle pain syndrome [31]. The current study found that continuous yoga reduced BW, decreased BFM, and increased muscle mass. However, yoga must be practiced on a regular basis until it is a daily habit. Effective strategies for weight loss require management strategies with a combination of dietary therapy and physical activity using behavioral interventions [32]. Yoga practices lead to an increase in energy expenditure, which results in statistically significant changes in body composition [33, 34]. The practice of only Hatha yoga for 30 min daily increased the metabolic equivalent of task (MET) to 2.5 in females [35].

The limitations of this study were the small sample size and the inclusion of only women, which limits the generalizability to men. Further research should be performed on male subjects or different age groups to determine the effect of this intervention. Our study reported only BMI, BFM, and muscle mass but not fat-free mass. We suggest that future work evaluate more accurate parameters, such as fat-free mass.

## 5. Conclusions

The current study demonstrated that the mean BMI and BFM of a yoga intervention group decreased significantly at week 8 and week 12. The muscle mass of the yoga group continued to improve at a rate of 0.515 per week. Multivariate logistic regression analysis confirmed that continuous yoga practice affected muscle mass. These findings demonstrated intervention effectiveness that was similar to other clinical research and indicated that continuous yoga practice may be used as an alternative therapy for obesity prevention and health promotion in adolescents with obesity.

## Figures and Tables

**Table 1:** Yoga curriculum.

FITT principle	Items	Details
F	3 times/week	Mon., Wed., Fri.
I	65–75% of maximum heart rate	135–145 times/min
T	50 min	Warm-up/stretching, 10 min yoga exercise, 12 poses, 30 min cooldown/relaxation, 10 min
T	Continuous yoga	Continuous yoga, 12 poses

FITT: frequency, intensity, time, and type.

**Table 2:** Demographic characteristics of the study participants.

Characteristics	Groups		*P* value
	Yoga (*n* = 20)	Control (*n* = 20)	
Weight (kg)	65.60 ± 10.15	67.53 ± 4.72	0.587
Height (cm)	164.25 ± 7.82	172.20 ± 6.61	>0.001^*∗*^
BMI (kg/m^2^)	22.70 ± 3.51	23.37 ± 1.63	0.433
BFM	29.25 ± 3.49	29.22 ± 3.86	0.968
Muscle mass	25.31 ± 1.60	24.25 ± 1.76	0.059

Data are presented as the means ± SD. ^*∗*^*P* < 0.05, significantly different compared to the control group.

**Table 3:** Body mass index (BMI) of the yoga and control groups.

Follow-up	Groups		*P* value^*∗*^
	Yoga (*n* = 20)	Control (*n* = 20)	
Week 0	22.70 (3.51)	23.37 (1.63)	0.433
Week 8	22.26 (3.37) †††	23.32 (1.61)	0.087
Week 12	22.02 (3.33) †††	23.42 (1.65)	0.018
Mean difference	0.681	0.058	NT
95% CI for difference	0.204–0.630	0.278–0.395	
*P* value^∗∗^	≤0.001	0.664	

Data are represented as the means (SD). NT: not detected. *Statistical analysis.*^*∗*^Mann–Whitney *U* test.^∗∗^Friedman's test. †Significant difference from day 0 within group (*P* ≤ 0.05).††Significant difference from day 0 within group (*P* ≤ 0.01). †††Significant difference from day 0 within group (*P* ≤ 0.001).

**Table 4:** Body fat mass of the yoga and control groups.

Follow-up	Groups		*P* value^*∗*^
	Yoga (*n* = 20)	Control (*n* = 20)	
Week 0	29.25 (3.49)	29.22 (3.86)	0.968
Week 8	27.49 (3.55) †††	28.68 (3.50) †	0.406
Week 12	26.37 (3.47) †††	29.02 (3.54)	0.013
Mean difference	2.879	0.200	NT
95% CI for difference	2.119–3.639	0.444–0.844	
*P* value^∗∗^	≤0.001	0.030	

Data are presented as the means (SD). NT: not detected. *Statistical analysis.*^*∗*^Mann–Whitney *U* test. ^*∗∗*^Friedman's test. †Significant difference from day 0 within group (*P* ≤ 0.05). ††Significant difference from day 0 within group (*P* ≤ 0.01). †††Significant difference from day 0 within group (*P* ≤ 0.001).

**Table 5:** Muscle mass of the yoga and control groups.

Follow-up	Groups		*P* value^*∗*^
	Yoga (*n* = 20)	Control (*n* = 20)	
Week 0	25.31 (1.60)	24.25 (1.76)	0.059
Week 8	25.39 (1.59)	23.62 (1.86) ††	0.005
Week 12	25.83 (1.45) ††	23.81 (1.83) †	0.002
Mean difference	0.515	−0.440	NT
95% CI for difference	0.130–0.900	(−0.888)–0.008	
*P* value^∗∗^	0.002	0.002	

Data are presented as the means (SD). NT: not detected. *Statistical analysis*. ^*∗*^Mann–Whitney *U* test. ^∗∗^ Friedman's test. †Significant difference from day 0 within group (*P* ≤ 0.05). ††Significant difference from day 0 within group (*P* ≤ 0.01). †††Significant difference from day 0 within group (*P* ≤ 0.001).

**Table 6:** Univariate and multivariate logistic regression analyses.

Characteristics	*n*	OR (95% CI)	*P* value	AOR (95% CI)	*P* value
Age	20.00 ± 0.75^*∗*^	1.20 (0.51–2.81)	0.670	0.71 (0.21–2.46)	0.590
BFM	29.23 ± 3.63^*∗*^	1.85 (1.27–2.71)	0.001^∗∗^	1.23 (0.78–1.95)	0.360
Muscle mass	24.77 ± 1.74^*∗*^	4.53 (1.75–11.69)	0.002^∗∗^	3.88 (1.08–13.93)	0.030^∗∗^
Yoga					
No	20 (50.00)	Ref.		Ref.	
Yes	20 (50.00)	0.81 (0.23–2.83)	0.750	0.39 (0.04–3.50)	0.400

^*∗*^Data are presented as the means ± SD. ^∗∗^Statistically significant at *P* < 0.05.

## Data Availability

The data used to support the findings of this study are available from the corresponding author upon request.
